# Automatic Prestimulation on Dairy Goats: Milking Efficiency and Teat-End Status

**DOI:** 10.3390/ani11010121

**Published:** 2021-01-08

**Authors:** Joel Bueso-Ródenas, Manuel Alejandro, Gema Romero, José Ramón Díaz

**Affiliations:** 1 Dpto. Producción Animal y Salud Pública, Universidad Católica de Valencia (UCV), C/ Guillem de Castro 94, 46001 Valencia, Spain; joel.bueso@ucv.es; 2 C/Anabel Segura, 7, 28108 Madrid, Spain; manuel_alejandromartinez@yahoo.com; 3 Dpto. Tecnología Agroalimentaria, Universidad Miguel Hernández (UMH), Ctra. de Beniel km 3.2, 03312 Orihuela, Spain; gemaromero@umh.es

**Keywords:** milk fractioning, Murciano-Granadina goats, mechanical prestimulation, teat condition

## Abstract

**Simple Summary:**

In the literature reviewed, there were no studies about how automatic mechanical stimulation affects milking efficiency and teat-end status in dairy goats. Three experiments were performed at the onset, middle, and end of lactation on Murciano-Granadina goats. In each experiment, milking with and without previous mechanical stimulation was tested. Milk fractioning, milking time, milk flows, and teat-end status assessed by ultrasonography and vacuum levels in the short milk tubes and short pulsation tubes were registered. Results showed that, conversely to dairy cows, investing in equipment for performing mechanical prestimulation in dairy goats is not needed, as it did not offer any advantage regarding the above mentioned variables.

**Abstract:**

Experiments carried out in dairy cows show that mechanical stimulation prior to milking offers a good release of oxytocin without involving changes in milk yield or a reduction of the milking time. The objective of the present study was to evaluate the effect of automatic prestimulation on milk fractioning, milking duration and milk flows, teat-end status, and vacuum levels at the short milk tubes and in the pulsation tubes of dairy goats. With this aim, three experiments in Latin square design were developed employing goats in different moments of the lactation: one of them at the onset of lactation, one at mid-lactation, and the last at the end of lactation. Two treatments were tested: milking with a mechanical prestimulation of 300 ppm for a 20-s period and milking without prestimulation. Results showed that prestimulation at the end of lactation showed slightly lower average milk flow (kg/min) values (0.53 ± 0.02 vs. 0.60 ± 0.02; *p* = 0.03) and lower maximum vacuum level values (Kpa) in the pulsation tubes (27.08 ± 0.15 vs. 39.48 ± 0.25; *p* < 0.01). No other differences were found in the variables related to milking efficiency or teat-end status in the three experiments carried out.

## 1. Introduction

Stimulation of the udder previous to milking is the common way to produce a good release of oxytocin and, therefore, entailing higher average milk flow, faster milkings, better emptying of the mammary gland, and better teat condition, improving the milking performance of the animals and, moreover, the optimal level of animal welfare [1]. Stimulation of the udder can be done by different methods: hand milking [2], suckling of the calf [3], or even vaginal stimulation [4]. The most common method of prestimulation includes forestripping of each teat and cleaning each teat with individual towels. As these operations involve investments in labor and time, some authors recommend that, in some situations, to achieve the greatest milking efficiency, clusters should be attached immediately without premilking stimulation [5].

Nowadays, in small ruminants, common milking routines include cluster attachment without preparation of the udder, machine milking, brief machine stripping, and cluster detachment [6]. Some authors [7,8] have even proposed elimination of the machine-stripping phase in those particular breeds selected for their milkability [9]. This reduction time would even be also enhanced with the implementation of automatic cluster removers [10].

As cisternal milk fraction of small ruminants can range from 40% in sheep [9] to 82% in goats [11] so, in these species, milking can begin without oxytocin release prior to milking [9]. In the other hand, lack of oxytocin-mediated milk ejection limits lactation persistency, potentially involving a loss of about 35% of the milk of the entire lactation [12]. It is also important in small ruminants to recuperate the last alveolar milk fraction, as it can contain up to 75% of the milk fat [12]. Recent studies carried out in Murciano-Granadina breed goats showed that daily values of residual milk in this breed can range from 112 [13] to 314 g [14]. Thus, any advance in getting a better milk yield from the animals could be decisive in the profitability of farms.

In recent decades, the level of automations implemented in milking machines has significantly increased [6,15,16,17]. In this line of work, milking machine manufacturers have implemented new generation pulsators with the option of practicing automatic stimulation of the teats. This automatic stimulation includes an elevation of the pulsation rate, up to 300 ppm for a period of 20–90 s, during the first part of the milking, just after milking units are attached [18]. The use of automatic prestimulation has been described as a favorable option to ensure a good release of oxytocin, improving the milking performance of dairy cows [19]. The use of automatic prestimulation in dairy cows can offer an increase in milk flow rate [20] and a reduction of machine-on time [21].

Experiments carried out in Saanen goats showed that manual prestimulation offered an enhancing of milk yield in late lactation and a daily reduction of 50 g in stripping yield in mid-lactation [22]. Another study found a delay in milk flow to reach 500 g/min after cluster attachment [23]. Another study performed in Alpine goats showed that manual prestimulation of 30 s resulted in higher average and peak flow rate, shortest milking time, highest milk yield, and lowest stripping [24]. Considering these latter results, automatic prestimulation in Murciano-Granadina goats can be a very profitable option to improve milking efficiency and reduce the residual milk fraction without time losses, as milkers did not have to handle the goats one by one, as would be the case in manual prestimulation.

To our knowledge, in the literature there are no experiments regarding automatic stimulation in dairy goats. The aim of this study was to evaluate the effect of automatic prestimulation on milking efficiency (milk fractioning, milking duration, and milk flows), teat-end status of dairy goats, and vacuum level at the short milk tube (maximum, minimum, average, and vacuum drops) of dairy goats during onset, mid-, and end of lactation.

## 2. Materials and Methods

### 2.1. Facilities and Animal Handling

The experiments were carried out at the research and teaching farm of the Escuela Politécnica Superior de Orihuela (Universidad Miguel Hernández de Elche, Spain). The Institutional Animal Care and Use Committee (IACUC) of this university approved the referenced Animal Use (UMH.DTA.JDS.001.09). A GeaFarm Technologies (Bönen, Germany) low line milking machine in a Casse 1 × 12 × 12 milking parlor was used. The parlor was equipped with a new-generation pulsator (StimoPuls Apex M, GeaFarm Technologies, Bönen, Germany), allowing automatic stimulation. Stimulation consisted of an elevation of the pulsation rate, up to 300 ppm for a 20-s period, during the first part of the milking, just after the milking units were attached. The milking units were equipped with TopFlow teatcups (GeaFarm Technologies, Bönen, Germany), short milk tubes with internal diameter of 10 mm, and 1-m -long milk tubes with internal diameter of 14 mm. Milking parameters were: system vacuum of 40 kPa, pulsation rate of 90 cycles/min, and pulsation ratio of 60:40. Milking was carried out once a day, as usual in this breed in Southeastern Spain, at 9:00 a.m. The milking routine included cluster attachment, machine milking, and automatic cluster removal at the end of the milking. If a teatcup falloff was observed during milking, it was immediately reattached. Finally, teats were immersed in an iodine solution (Proactive, DeLaval, Tumba, Sweden). Automatic cluster removers were programmed with a minimum milking time of 50 s, a milk flow threshold of 150 g/min, and a delay time of 10 s [25]. No manual stimulation for cleaning procedures previous to cluster attachment was performed.

### 2.2. Experimental Design

Three identical experiments were carried out as three distinct Latin squares: one of them at the onset of lactation (3rd ± 1 week post-partum), one at mid-lactation (18th ± 1 week post-partum), and the other at the end of lactation (29th ± 1 week post-partum). The duration of each experiment was eight days (two periods of four days’ duration) and the animals enrolled in each experiment were different: *n* = 68 goats (*n* = 52 multiparous and *n* = 16 primiparous) in the first experiment, *n* = 46 goats (*n* = 34 multiparous and *n* = 12 primiparous) in the second, and *n* = 42 goats (*n* = 34 multiparous and *n* = 8 primiparous) in the third. The average weight of the goats was 50 kg and parities ranged from 1 to 5. Goats during lactation were fed twice a day with 1500 g/day per animal of cereal mixture (corn 23%, barley 21%, beet pulp dried 14%, oat groats 13%, soybean meal 9.2%, peas 8%, sunflower seeds 3.3%, sunflower meal 3%, orange pulp dried 4.8%, salt 0.4%, and soybean oil 0.3%; DM (Dry matter) 87.2%) and 1000 g/day per animal of alfalfa hay (DM 89.3%). Straw and water were offered ad libitum. No pregnant goats were used in these experiments.

**Figure 1:**
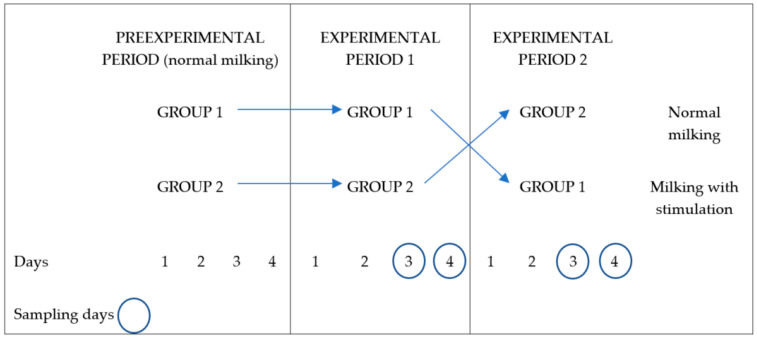
Schedule of each of the three experiments developed at the onset of lactation, at mid-lactation, and at the end of lactation according to the type of milking and the period within each experiment.

Pre-experimental sampling was carried out beforehand to know the pre-experimental conditions and select the goats enrolled in each of the three experiments. The variables recorded were milking duration, machine milk, hand stripping milk, residual milk, maximum and average milk emission flow, and udder health status (see Section 2.3). The goats selected had a milk yield of over 1 kg at the onset and mid-lactation or 0.5 kg at the end of lactation, respectively, with milking time lower than 6 min, and without clinical mastitis. Milk yield and milking duration (mean ± sd) of the goats selected for each experiment were, respectively, 3.06 ± 0.77 kg and 246 ± 85 s at the onset of lactation, 2.19 ± 0.51 kg and 221 ± 57 s at mid-lactation, and, finally, 1.26 ± 0.31 kg and 151 ± 45 s at the end of lactation. Selected animals were split into two groups with similar characteristics in terms of parity, machine milk yield, milking time, and udder health status (same number of subclinical mastitis animals in every group). Before each experiment, in order to acclimate the animals to the new group and pen, both groups were milked with normal milking during a four-day acclimation period. Later, during the experimental period, each group was milked with a treatment (normal milking or milking with automatic prestimulation) for four days: the first two days to accustom the animals to the treatment (adaptation period), a third day to record milking efficiency variables (sampling day), and the fourth day to record teat end condition variables (sampling day), whereupon animals were switched to another treatment and the procedure was repeated (Figure 1).

### 2.3. Variables Analyzed

#### 2.3.1. Efficiency of Milking (Milk Fractioning, Milking Duration, and Milk Flows)

Milking routine during this sampling day included: attaching the teatcups, automatic prestimulation according to experimental design, machine milking (recording of machine milk and milking duration), automatic cluster removal, hand stripping (recording hand stripping milk), and recording of residual milk after application of 4 IU oxytocin (Dalmatocina^®^, Fatro Ibérica, Barcelona, Spain). Finally, teats were immersed in an iodine solution. Machine milk (kg) was the amount milked in the period from teatcup attachment to teatcup detachment, carried out by an experienced milker. This variable and average (kg/min) and maximum (kg/min) milk flows during the main milking phase were registered using Lactocorder^®^ devices (Lactocorder, Balgach, Switzerland). Milking duration (min) was the time required to obtain machine milk and was registered using a digital chronometer (HS–70W, Casio^®^, Tokyo, Japan). Hand stripping milk (g) and residual milk (g) were weighed with a digital device with ± 1 g precision (BC-200, Fagor^®^, Mondragón, Spain).

#### 2.3.2. Sanitary Status of the Mammary Gland

Milk samples were taken by hand from each mammary gland before milking. For bacteriological analysis, foremilk was discarded, the teats were cleaned with 70% alcohol, and 5-mL samples of milk were taken. For somatic cell count (SCC) analysis, 50-mL samples of milk were taken and azidiol was added to them. The evaluation of the mammary gland health status of the goats in the three pre-experimental periods was developed to include the same number of animals with subclinical mastitis in both groups according the procedures of Bueso-Ródenas et al. [13].

#### 2.3.3. Teat-End Status

To study the teat-end status of the goats, a portable ultrasound device (Agroscan AL, ECM, Noveko International Inc., Angoulême, France) was used. During the experiment performed at the onset of lactation, ultrasound scans of the right teat of each animal were performed before and after milking with a linear probe at 5 MHz frequency and 8 cm depth [26]. The sonographic images obtained were recorded and digitally scanned with Real DVD Studio Gold software (NPG Technology, Madrid, Spain). Later, video editor software (Ulead Video Studio, Corel, Ottawa, ON, Canada) was used to take a frame of the sagittal plane of the teat end at the level of the teat sphincter. Finally, another software suite (Ecopezón, Universidad Miguel Hernández de Elche, Elche, Spain) was used to measure the following variables: (1) teat wall thickness (TWT, cm): two lines (lines A and B) were traced along the middle axis of the two teat walls and at 1 cm a line was drawn perpendicular to it, measuring the width of each wall (upper and lower) to obtain the mean value of both measurements; (2) teat-end area (TEA, cm^2^): area from the inner to the outer end of the teat canal; (3) teat wall area (TWA, cm^2^): area covering both walls (upper and lower) to a distance of 1 cm from the teat end, including the teat-end area; (4) teat canal length (TCL, cm): distance from the inside to the outside of the teat canal. From the values recorded in the scans before the cluster attachment and after their removal, the percentage increase of each variable was computed according to the following mathematical formula: Increase % = ((Value after milking–Value before milking)/ Value before milking)) × 100. The new variables were: increase of teat wall thickness (ITWT, %), increase of teat wall area (ITWA, %), increase of teat-end area (ITEA, %), and, increase of teat canal length (ITCL, %).

#### 2.3.4. Vacuum Level Variables and Pulsation Tests

During the experiment performed at the onset of lactation, the variables related to the variation of vacuum level were recorded using a Vadia^®^ device (Biocontrol^®^, Rakkestad, Norway). Twenty vacuum level measurements within each of the groups were recorded in random goats. The vacuum level measurements were performed in the short milk tubes (SMT) and in the short pulsation tubes (SPT) during (1) the first 20 s after the cluster attachment, which included the stimulation phase in the control group, and (2) the main milking phase in both groups. Variables recorded were maximum vacuum level (kPa), average vacuum level (kPa), and minimum vacuum level (kPa). From the values of maximum vacuum level, average vacuum level and minimum vacuum level during measurements in the SMT and in the SPT, vacuum drop in the SMT (maximum vacuum level in the SMT—minimum vacuum level in the SMT, kPa), difference in maximum vacuum level (DifMaxVL, maximum vacuum level in the SMT—maximum vacuum level in the SPT), and difference in average vacuum level (DifAvgVL, average vacuum level in the SMT—average vacuum level in the SPT) were calculated. These previous two variables were used as an estimation of the vacuum level difference between both parts of the liner. During the experiment performed at the onset of lactation, the pulsation tests were carried out during (1) stimulation and (2) normal milking with a Pulsotest Comfort (GeaFarm Technologies, Bönen, Germany) device (response rate of 1500 kPa/s when directly connected to the pulsator [27] and set at a sampling rate frequency of 166.7 Hz). Variables recorded were duration of A-phase duration, of B-phase duration, of C-phase, and duration of D-phase (all of them expressed as percentage of the pulsation cycle and time in ms).

### 2.4. Statistical Analysis

To determine whether there were differences between the groups of animals at the onset of the three experiments, a general linear model was applied (Proc. GLM, SAS 9.2., 2012). Dependent variables were milking duration, machine milk, hand stripping milk, residual milk, and maximum and average milk emission flow. The fixed effect considered was the animal group (1 or 2). For each experiment, the relationship between the variables related to milking efficiency (milking duration, machine milk, hand stripping milk, residual milk, and maximum and average milk emission flow) and teat-end condition (ITWT, ITWA, ITEA, ITCL) with the treatment applied (with automatic stimulation or without it) was studied using a general mixed model (Proc. Glimmix, SAS 9.2., 2012). Fixed effects considered were the treatment and the period in each experiment (period one and period two). For milking duration, the machine milk was added as covariable. Interaction between the period in each experiment and the treatment was not significant for any variable and was discarded from the final model. For every variable, the individual goat was considered the random term. The model using this hierarchical structure provided the best fit for the data when compared to different models considering other covariance and hierarchical structures (as assessed using Bayesian and Akaike information criteria). The effect of the treatment on the maximum, minimum, and average vacuum levels in SMT and SPT, vacuum drops, DifMaxVL, and DifAvgVL was analyzed using a general linear mixed model (Proc. GLM, SAS, 9.2., 2012), considering the treatment applied (with automatic stimulation or without it), the moment at which measurements were taken (first 20 s after cluster attachment or main milking phase), and the interaction between both, as fixed effects. Finally, the effect of the treatment on the pulsation tests’ variables (duration of A-phase, duration of B-phase, duration of C-phase, and duration of D-phase; all of them expressed as percentage of the pulsation cycle and time in ms) was analyzed using a general linear mixed model (Proc. GLM, SAS, 9.2., 2012), considering the treatment applied (with automatic stimulation or without it) as fixed effect.

## 3. Results

There were no significant differences between groups of animals during the three pre-experimental periods for any of the variables analyzed (milking duration, machine milk, hand stripping milk, residual milk, maximum and average milk emission flows, *p* > 0.05). The evaluation of the mammary gland health status of the goats in the three pre-experimental periods showed that at the beginning of lactation there were *n* = 6 goats with subclinical mastitis (three per group), *n* = 4 in mid-lactation (two per group), and *n* = 4 at the end of lactation (two per group). During the experimental periods, the statistical analysis showed that at the beginning of lactation there were no effects of the treatment or the period in each experiment (period one or period two) on the milk fractioning, milking duration, or milk flows (*p* > 0.05). In mid-lactation, the period in each experiment had an effect on milking duration (*p* = 0.02) and average milk flow (*p* = 0.03). At the end of lactation, the treatment applied had an effect on average milk flow (*p* = 0.04). Thus, values of the variables regarding milking efficiency did not differ among treatments during the onset of lactation or in mid-lactation. The only differenc. was at the end of lactation, where the milking with automatic previous stimulation showed significant lower values of average milk flow with differences up to 0.07 kg/min (0.53 ± 0.02 vs 0.60 ± 0.02, Table 1). Notwithstanding this fact, values for milking duration and machine milk were similar (147 ± 10 s vs. 157 ± 10 s and 1.24 ± 0.06 kg vs. 1.22 ± 0.06 kg, respectively). Moreover, none of the effects studied was significant (*p* > 0.05) on teat-end status variables (ITWT, ITEA, ITWA, ITCL); thus, values of these variables did not differ among treatments (Table 2).

Regarding variables related to vacuum levels in the SMT, none of the effects studied affected the average vacuum level and maximum vacuum level variables, so no differences between milking treatments or times were found. However, during the 20 s of stimulation, higher values for minimum vacuum level (difference of 3.81 KPa) were found, which implied that during stimulation the vacuum drop values (difference of 3.83 KPa) were lower. A similar situation was shown in the vacuum levels of the SPT. During stimulation, average and maximum vacuum levels in pulsation tubes were significantly lower than in the other treatments or times, with differences of 9.5 and 12.4 KPa, respectively. The most relevant results were found when the differences in vacuum level between the two parts of the liner were calculated. Previous stimulation offered much higher significant values of DifMaxVL and DifAvgVL (difference of 12.3 and 9.55 KPa, respectively, Table 3).

Values of the pulsation tests revealed that stimulation at 300 cycles/min, compared to normal milking at 90 cycles/min, affected not only duration of the pulsation phases expressed in ms, as expected, but also the corresponding percentage of each phase (A, B, C, D) in the single pulsation cycle. Regarding the complete pulsation cycle (100%) A-phase during stimulation showed an absolute difference of +5.5%; B-phase, −1%; C-phase, +8.2%; and D-phase, −12.7%. This last result implied that D-phase during stimulation was shortened to only 28.57 ms (179.18 ms in normal milking, Table 4).

## 4. Discussion

Milk yields observed in this study agree with those observed in other studies conducted in the same goat breed in similar conditions [13]. Machine milk was not affected by the stimulation in any of the experiments performed. This fact is consistent with the experiments carried out in cows [4,5,20].

Despite the considerable benefits described in Alpine goats in terms of milking efficiency and milk fractioning [24], in the present study there was no positive effect of the stimulation on these variables. In another previous study also carried out in goats [22], practicing manual stimulation in mid-lactation, there was a decrease of stripping milk (0.15 vs. 0.10 kg, normal milking and manual prestimulation, respectively) but no differences were found at the onset and end of lactation in other variables. These latter authors suggested that release of oxytocin was not important for milk removal, but observed an increase in oxytocin during the stripping phase. It seems that the variability of the procedures carried out during the previous stimulation (manual vs. automatic) and the different milkability of the breeds (Alpine, Saanen, and Murciano-Granadina) could have had an influence on the results. For now, it is unclear whether stripping at the end of milking is necessary or not in goat milking, and the results suggest that there are big differences between breeds [28]. Studies conducted in Murciano-Granadina goats, comparing routines including machine stripping and automatic cluster removal without stripping, showed no differences in milk fractioning, milk composition, or milk yield persistency throughout lactation [25]. Meanwhile, Canary breed goats have large stripping milking fractions (up to 0.69 kg) and, including the stripping phase in the routine, may have economic significance for the profitability of farms [29]. In any case, although dairy goats store a great percentage of their milk in the gland cistern, and, after cluster attachment, milk is available from the very beginning of the milking process, the release of oxytocin is mandatory to achieve the maximum yield and fat from the udder, minimizing the amount of residual milk [4,12]. Moreover, this oxytocin release would produce a second peak of milk flow and, if the oxytocin release happens while the cisternal fraction has not been completely removed, the cited second milk peak would happen even earlier and the two peaks would be overlapped [9], thus increasing milking efficiency. So, although in the present experiment automatic prestimulation offered no advantages on the milking of Murciano-Granadina goats, the benefits of an early release of oxytocin should be considered when developing the milking routine, including calmed surrounding and gentle handling of the animals.

According to ISO standards for small ruminants’ milking [30], B-phase should be longer than 30% of the pulsation cycle, C-phase shorter than A-phase, and D-phase longer than 15%. In this sense, prestimulation cycles would breach this last standard, considering the shortening of the D-phase observed during prestimulation compared to that of normal milking (14.34% vs. 27.01%, respectively). This fact could have limited the massage effect of the liner, potentially causing more edema in the teat end, as has been described in cows [31]. However, this shortening had no effect on the variables related to teat-end status after milking. Until now, only milking without automatic cluster removers [32] or extreme situations such as forced overmilking or old and twisted liners [33] have been factors affecting teat-end status in small ruminants. The lack of any significant effect of the treatments on the variables related to teat-end status is also linked to the similarity of the values for milking duration and average vacuum level at the teat end.

In their experiments on prestimulation in dairy cows, Weiss and Bruckmaier [20] used different maximum pulsation vacuum levels. These authors found that when applying a low maximum pulsation vacuum during prestimulation (17 KPa), the milk yield recorded during this prestimulation period was lower than when high maximum pulsation vacuum was applied (30 KPa). In the present experiments, maximum pulsation vacuum recorded during prestimulation was lower (27.08 KPa) than in the control group (39.34 KPa), which would have limited the extraction of milk during the prestimulation time. At the beginning of lactation and in mid-lactation, this fact was diluted by greater machine milk and longer milking duration, but at the end of lactation, when the total milk available and the milking duration are lower, the variable average milk flow was negatively affected. It is possible that, during this previous stimulation, due to the difference in maximum vacuum between the two parts of the liner (12.59 KPa), milking is not so effective, as liners are not completely open during the milking phase of the pulsation cycles.

Vacuum level values in the SMT were similar among treatments and similar to those observed in experiments carried out in this breed and milking conditions (40 KPa system vacuum and low-line installation) [25]. Although the present study was performed as three short-term experiments and a long-term experiment during a lactation is needed to confirm this hypothesis, according to previous results of this research group [13], vacuum drops recorded and the abovementioned lack of significant differences in variables related to teat-end status, automatic prestimulation would not involve risks for the mammary gland health status.

## 5. Conclusions

Milking with previous automatic stimulation in dairy goats did not show any advantage in enhancing the milking efficiency. Moreover, it offered lower average milk flow values during the end of lactation of the animals without consequences in terms of milking duration or in milk extracted. In view of the results of this study, including pulsators with these features when installing milking machines for dairy goats could be a questionable additional investment.

## Figures and Tables

**Table 1:** Effect of the treatment (normal milking vs. milking with automatic prestimulation) on variables related to milking efficiency in Murciano-Granadina goats on three moments of the lactation (least square means ± standard error of the mean).

Variable	Normal Milking	Automatic Prestimulation	SEM	Significance Level
Experiment 1: Onset of lactation				
Machine Milk (Kg)	3.05	3.09	0.11	0.44
Hand Stripping Milk (g)	205	213	27	0.72
Residual Milk (g)	207	219	20	0.53
Milking Duration (s)	252	251	13	0.91
Average Milk Flow (Kg/Min)	0.87	0.87	0.03	0.68
Maximum Milk Flow (Kg/Min)	1.17	1.17	0.05	0.89
Experiment 2: Mid-lactation				
Machine Milk (Kg)	2.18	2.17	0.1	0.95
Hand Stripping Milk (g)	191	158	36	0.39
Residual Milk (g)	129	129	19	0.98
Milking Duration (s)	223	229	12	0.58
Average Milk Flow (Kg/Min)	0.73	0.70	0.03	0.35
Maximum Milk Flow (Kg/Min)	1.01	0.99	0.04	0.68
Experiment 3: End of Lactation				
Machine Milk (Kg)	1.24	1.22	0.06	0.58
Hand Stripping Milk (g)	85	73	13	0.24
Residual Milk (g)	125	101	17	0.58
Milking Duration (s)	147	157	10	0.67
Average Milk Flow (Kg/Min)	0.60 ^a^	0.53 ^b^	0.02	0.03
Maximum Milk Flow (Kg/Min)	0.83	0.87	0.03	0.07

Values in the same line with different letters (^a, b^) differ at *p* < 0.05.

**Table 2:** Effect of the treatment (normal milking vs. milking with automatic prestimulation) on variables related to teat-end status in Murciano-Granadina goats on the onset of lactation (least square means ± standard error of the mean).

Variable	Normal Milking	Automatic Prestimulation	Significance Level
ITWT (%)	40.36 ± 2.14	43.44 ± 2.12	ns
ITEA (%)	16.11 ± 1.11	15.82 ± 1.10	ns
ITWA (%)	30.73 ± 3.31	31.36 ± 3.27	ns
ITCL (%)	21.14 ± 2.38	24.20 ± 2.36	ns

ITWT: increase in teat wall thickness; ITEA: increase in teat-end area; ITWA: increase in teat wall area; ITCL: increase in teat canal length; ns: not significant, *p* > 0.05.

**Table 3:** Effect of the treatment (normal milking vs. milking with automatic prestimulation) during two moments of the milking (first 20 s and main milking phase) on variables related to vacuum level during the mechanical milking of Murciano-Granadina goats on the onset of lactation (least square means ± standard error of the mean).

	Treatment	Normal Milking		Automatic Prestimulation		
		Moment		Moment		
Variable		First 20 s	Main Milking Phase	First 20 s	Main Milking Phase	Significance Level
AvgVLSMT		38.27 ± 0.15	38.35 ± 0.11	38.45 ± 0.11	38.41 ± 0.06	ns
MinVLSMT		32.23 ± 1.35 ^a^	31.05 ± 0.95 ^a^	34.86 ± 0.88 ^b^	32.35 ± 0.49 ^a^	<0.01
MaxVLSMT		39.81 ± 0.11	39.68 ± 0.08	39.66 ± 0.07	39.75 ± 0.04	ns
Vac Drop SMT		7.5 ± 1.35 ^a,b^	8.62 ± 0.96 ^b^	4.79 ± 0.88 ^a^	7.4 ± 0.49 ^b^	<0.01
AvgVLPulsation		23.10 ± 0.12 ^a^	23.15 ± 0.08 ^a^	13.65 ± 0.07 ^b^	23.13 ± 0.04 ^a^	<0.01
MinVLPulsation		0.01 ± 0.11 ^a^	0.01 ± 0.08 ^a^	0.98 ± 0.07 ^b^	0.01 ± 0.04 ^a^	<0.01
MaxVLPulsation		39.48 ± 0.25	39.47 ± 0.18	27.08 ± 0.15	39.34 ± 0.08	<0.01
Average VDiff		15.28 ± 0.15 ^a^	15.26 ± 0.23 ^a^	24.81 ± 0.54 ^b^	15.34 ± 0.46 ^a^	<0.01
Maximum VDiff		0.31 ± 0.17 ^a^	0.29 ± 0.18 ^a^	12.59 ± 1.41 ^b^	0.42 ± 0.21 ^a^	<0.01

AvgVLSMT: average vacuum level in the short milk tubes; MaxVLSMT: maximum vacuum level in the short milk tubes; MinVLSMT: Minimum Vacuum Level in the Short Milk Tubes; Vac Drop SMT: Vacuum Drop in the Short Milk Tubes (maximum–minimum vacuum levels in the short milk tubes) AvVLPulsation: average vacuum level in pulsation tubes; MaxVLPulsation: maximum vacuum level in pulsation tubes; MinVLPulsation: minimum vacuum level in pulsation tubes; VDiff: vacuum difference between short milk tubes and short pulsation tubes (estimation of the vacuum difference between both parts of the liners); values in the same line with different letters (^a, b^) differ at *p* < 0.01. Total measurements: 80.

**Table 4:** Effect of the treatment (normal milking, 90 cycles per min, vs. milking with automatic prestimulation, 300 cycles per min) on the pulsation phases during the mechanical milking of Murciano-Granadina goats on the onset of lactation (least square means ± standard error of the mean).

Pulsation Phases	Normal Milking	Automatic Prestimulation	Significance Level
A-phase duration (%)	17.12 ± 0.24 ^a^	22.67 ± 0.22 ^b^	<0.01
A-phase duration (ms)	113 ± 1.35 ^a^	45.23 ± 1.25 ^b^	<0.01
B-phase duration (%)	43.36 ± 0.25 ^a^	42.31 ± 0.23 ^b^	<0.01
B-phase duration (ms)	287.95 ± 1.36 ^a^	84.38 ± 1.25 ^b^	<0.01
C-phase duration (%)	12.54 ± 0.45 ^a^	20.74 ± 0.42 ^b^	<0.01
C-phase duration (ms)	83.27 ± 1.45 ^a^	41.38 ± 1.36 ^b^	<0.01
D-phase duration (%)	27.01 ± 0.63 ^a^	14.34 ± 0.58 ^b^	<0.01
D-phase duration (ms)	179.18 ± 1.74 ^a^	28.57 ± 1.61 ^b^	<0.01

Values in the same line with different letters (^a, b^) differ at *p* < 0.01. Total measurements: 80.

## Data Availability

The data presented in this study are available on request from the corresponding author.

## References

[1] Bruckmaier R.M., Wellnitz O. (2007). Induction of milk ejection and milk removal in different production systems. J. Anim. Sci..

[2] Gorewit R.C., Svenernersten K., Butler W.R., Uvnäs-Moberg K. (1992). Endocrine responses in cows milked by hand and machine. J. Dairy Sci..

[3] Lupoli B.B., Johansson K., Uvnas-Moberg K., Svennersten-Sjaunja K. (2001). Effect of suckling on the release of oxytocin, prolactin, cortisol, gastrin, cholecystokinin, somatostatin, and insulin in dairy cows and their calves. J. Dairy Sci..

[4] Bruckmaier R.M., Blum J.W. (1998). Oxytocin release and milk removal in ruminants. J. Dairy Sci..

[5] Edwards J.P., Jago J.G., Lopez-Villalobos N. (2013). Short–term application of prestimulation and increases automatic cluster remover threshold affect milking characteristics of grazing dairy cows in late lactation. J. Dairy Sci..

[6] Bueso-Ródenas J., Romero G., Arias R., Rodríguez A.M., Díaz J.R. (2015). Effect of automatic cluster removers on milking efficiency and teat condition of Manchega ewes. J. Dairy Sci..

[7] Knight T.W., Gosling L.S. (1995). Effects of milking frequency and machine–stripping on the yield and composition of milk from Poll Dorset ewes. N. Z. J. Agric. Res..

[8] McKusick B.C., Thomas D.L., Berger Y.M. (2003). Effect of omission of machine stripping on milk production and parlor throughput in East Friesian dairy ewes. J. Dairy Sci..

[9] Marnet P.G., McKusick B.C. (2001). Regulation of milk ejection and milkability in small ruminants. Livest. Sci..

[10] Tangorra F.M., Costa A., Guidobono-Cavalchini A. Preliminary results of a field study on goats milk yield and lactation persistency as affected by automatic cluster removals. Proceedings of the International Conference, SHWA2010.

[11] Torres A., Castro N., Hernández-Castellano L.E., Argüello A., Capote J. (2013). Short communication: Effects of milking frequency on udder morphology, milk partitioning, and milk quality in 3 dairy goat breeds. J. Dairy Sci..

[12] Labussiere J. (1988). Review of physiological and anatomical factors influencing the milking ability of ewes and the organization of milking. Livest. Sci..

[13] Bueso-Ródenas J., Romero G., Navarro A., Perez E., Díaz J.R. (2019). Effect of the pulsation type (alternate or simultaneous) on milk yield and health status of the mammary gland of Murciano-Granadina goats. J. Dairy Sci..

[14] Fernández N., Martí J.V., Rodríguez M., Peris C., Balasch S. (2020). Machine milking parameters for Murciano-Granadina breed goats. J. Dairy Sci..

[15] Magliaro A.L., Kensinger R.S. (2005). Automatic cluster remover settings affects milk yield and machine–on time in dairy cows. J. Dairy Sci..

[16] Ait-Saidi A., Caja G., Carné S., Salama A.A., Ghirardi J.J. (2008). Comparison of manual versus semiautomatic milk recording systems in dairy goats. J. Dairy Sci..

[17] Alejandro M. (2016). Automation devices in sheep and goat machine milking. Small Rumin. Res..

[18] Khatun M., Thomson P.C., Kerrisk K.L., Lyons N.A., Clark C.E.F., Molfino J., Garcia S.C. (2018). Development of a new clinical mastitis detection method for automatic milking systems. J. Dairy Sci..

[19] Karch G., Worstorff H., Prediger A., Reinhardt F. (1988). Stimulation capacity of the vibration system with special regard to the type of inflation. Milchwissenschaft.

[20] Weiss D., Bruckmaier R.M. (2005). Optimization of individual prestimulation in dairy cows. J. Dairy Sci..

[21] Watters R.D., Bruckmaier R.M., Crawford H.M., Schuring N., Schukken Y.H., Galton D.M. (2015). The effect of manual and mechanical stimulation on oxytocin release and milking characteristics in Holstein cows milked 3 times daily. J. Dairy Sci..

[22] Bruckmaier R.M., Ritter C., Schams D., Blum J.W. (1994). Machine milking of dairy goats during lactation, udder anatomy, milking characteristics, and blood concentrations of oxytocin and prolactin. J. Dairy Res..

[23] Romero G., Panzalis R., Ruegg P. (2017). Relationship of goat milk flow emission variables with milking routine, milking parameters, milking machine characteristics and goat physiology. Animal.

[24] Basic Z., Dzidic A., Kostelic A. (2009). The effect of prestimulation on milking characteristics during machine milking of goat. Mljekarstvo.

[25] Bueso-Rodenas G., Romero G., Roca A., Díaz J.R. (2014). Effect of One Automatic Cluster Remover (ACR) Setting on Milking Efficiency on Murciano-Granadina Goats. Livest. Sci..

[26] Díaz J.R., Alejandro M., Peris C. (2013). Use of ultrasound scanning to estimate teat wall thickness in Murciano-Granadina goats. Livest. Sci..

[27] Rasmussen M.D., Reinemann D.J., Mein G.A. (2003). Measuring vacuum in milking machines. IDF Bull..

[28] Dzidic A., Rovai M., Poulet J.L., Leclerc M., Marnet P.G. (2019). Review, Milking routines and cluster detachment levels in small ruminants. Animal.

[29] Capote J., Argüello A., Castro N., López J.L., Caja G. (2006). Short communication, Correlations between udder morphology, milk yield, and milking ability with different milking frequencies in dairy goats. J. Dairy Sci..

[30] ISO Standard 5707:2007 (2007). Milking Machines Installations-Construction and Performance.

[31] Upton J., Penry J.F., Rasmussen M.D., Thompson P.D., Reinemann D.J. (2016). Effect of pulsation rest phase duration on teat end congestion. J. Dairy Sci..

[32] Romero G., Bueso-Ródenas J., Gascó P., Díaz J.R. (2015). Effect of the automatic cluster removers (ACRs) in the milking of Murciano–Granadina goats during lactation. Small Rumin. Res..

[33] Alejandro M., Roca A., Romero G., Díaz J.R. (2014). Effects of overmilking and liner type and characteristics on teat tissue in small ruminants. J. Dairy Res..

